# The role of modern agricultural technologies in improving agricultural productivity and land use efficiency

**DOI:** 10.3389/fpls.2025.1675657

**Published:** 2025-09-16

**Authors:** Xie Chen

**Affiliations:** Department of Economics, Hunan Agricultural University, Changsha, Hunan, China

**Keywords:** precision agriculture, biotechnology, smart irrigation, vertical farming, AI- sustainability, food security

## Abstract

Modern agricultural technologies are crucial for addressing global food security and environmental sustainability challenges amidst a growing population and climate change. These innovations, including precision agriculture, biotechnology, smart irrigation, automation, vertical farming, and artificial intelligence (AI), significantly enhance productivity and land use efficiency. Precision agriculture, utilizing GPS, drones, and IoT, improves yields by 20–30% and cuts input waste by 40–60%. Biotechnology, with CRISPR and GMOs, delivers drought and pest-resistant crops, stabilizing yields, as seen with Bt cotton reducing pesticide use by 50% in India. Smart irrigation boosts water efficiency by 40–60%, while automation and robotics mitigate labor shortages and reduce costs by 25%. Vertical farming increases yields 10–20 times with 95% less land and water, supporting urban food security. AI analytics enhance decision-making with over 90% accuracy in forecasting and resource allocation. Despite these benefits, high costs, technological illiteracy, and regulatory issues hinder adoption, especially among smallholders. Policy support, public-private partnerships, and training are vital for broader technology access and fair benefits. Integrating renewable energy and circular economy principles into aggrotech presents a path to sustainability. This review highlights the transformative potential of modern technologies for sustainable intensification, increasing productivity without expanding farmland, while lessening environmental impacts. It underscores the need for coordinated efforts to overcome adoption challenges and harness these innovations for global food security and climate resilience.

## 1 Introduction

The global agricultural sector faces mounting pressures from population growth, climate change, and land degradation, threatening long-term food security. By 2050, the world’s population is expected to reach 9.7 billion, requiring a 70% increase in food production to meet demand. However, climate change has disrupted traditional farming systems through rising temperatures, erratic rainfall, and extreme weather events, reducing crop yields by up to 30% in vulnerable regions. Simultaneously, land degradation, driven by deforestation, soil erosion, and overuse of agrochemicals, has rendered 33% of global soils unproductive (Naik et al., 2025). These challenges are compounded by urbanization, which consumes arable land at an estimated 1.5 million hectares annually (Hu et al., 2025). Smallholder farmers, who produce 80% of food in developing countries, are disproportionately affected due to limited access to resources (Touch et al., 2024). Without intervention, these trends could exacerbate hunger, poverty, and ecosystem collapse, particularly in sub-Saharan Africa and South Asia (Otekunrin, 2025). Addressing these issues requires sustainable intensification, increasing yields without expanding farmland, through technological innovation. Modern agricultural technologies offer a viable pathway by optimizing inputs, reducing waste, and adapting to environmental stressors (Şimşek, 2025). However, widespread adoption remains hindered by economic, social, and infrastructural barriers, necessitating coordinated policy and investment.

Improving agricultural productivity (output per unit of input) and land use efficiency (yield per hectare) is critical to feeding a growing population sustainably. Traditional farming methods, reliant on extensive land use and chemical inputs, are increasingly inefficient and ecologically harmful (Tilman et al., 2011). For example, conventional irrigation wastes 50-60% of water due to evaporation and runoff (Jägermeyr et al., 2015), while excessive fertilizer use contributes to nitrogen pollution and dead zones in aquatic ecosystems (Diaz and Rosenberg, 2008). A study in Thailand demonstrated that precision agriculture, leveraging GPS, drones, and IoT sensors, increased dairy herd productivity by optimizing feed and resource allocation (Sarttra and Kiatcharoenpol, 2025). Similarly, in Ethiopia, a multistakeholder approach to lentil varietal adoption has shown significant improvements in food security and livelihoods (Najjar et al., 2025). These innovations have not only stabilized yields but also improved farmers’ incomes. By contrast, precision agriculture leverages GPS, sensors, and data analytics to apply water and nutrients only where needed, boosting yields by 20-30% while reducing resource waste (Gebbers and Adamchuk, 2010). Similarly, vertical farming produces 10–20 times more crops per square meter than traditional fields (Kozai and Niu, 2016), offering a solution for urban food deserts. Enhanced land use efficiency also mitigates deforestation, which accounts for 10% of global CO_2_ emissions (Searchinger et al., 2018). Furthermore, climate-smart crops, such as drought-resistant maize and flood-tolerant rice, stabilize yields in extreme weather (Ray et al., 2019). Without these innovations, meeting the UN Sustainable Development Goals (SDGs), particularly Zero Hunger (SDG 2) and Responsible Consumption (SDG 12), will be unattainable (UN, 2015). Thus, investing in modern agri-tech is not just an economic imperative but a moral and environmental necessity (Foley et al., 2011).

Modern agricultural technologies encompass a diverse suite of innovations designed to enhance efficiency, sustainability, and resilience. Precision agriculture utilizes GPS-guided tractors, drones, and satellite imagery to monitor crop health, soil conditions, and moisture levels in real time (Gebbers and Adamchuk, 2010). These tools enable variable-rate application (VRA) of inputs, reducing fertilizer and pesticide overuse by 40-60% (Zhang et al., 2022). Biotechnology, including genetically modified organisms (GMOs) and CRISPR gene editing, has produced crops resistant to pests, diseases, and climate stressors (Jinek et al., 2012). For instance, Bt cotton reduces pesticide use by 50% while increasing yields (Qaim, 2020). Internet of Things (IoT) devices, such as soil sensors and automated irrigation systems, optimize water use by adjusting delivery based on real-time data (Boursianis et al., 2022). Automation and robotics, like autonomous harvesters and robotic weeders, address labor shortages and cut production costs by 25% (Lowenberg-DeBoer et al., 2020). Controlled Environment Agriculture (CEA), including hydroponics and vertical farms, enables year-round production with 95% less water than conventional methods (Kozai and Niu, 2016). Artificial intelligence (AI) further enhances decision-making through predictive analytics, forecasting pest outbreaks and yield trends (Kamilaris et al., 2017). Together, these technologies form an integrated, data-driven farming ecosystem capable of addressing 21st-century challenges (Wolfert et al., 2017).

This review aims to systematically evaluate the role of modern agricultural technologies in improving productivity and land use efficiency while identifying barriers to adoption. First, it synthesizes empirical evidence on yield enhancements from precision farming, biotechnology, and automation, drawing on meta-analyses and case studies (e.g., Zhang et al., 2022; Lowenberg-DeBoer et al., 2020). Second, it assesses land-saving potential through innovations like vertical farming and no-till agriculture, quantifying reductions in deforestation and soil degradation (Searchinger et al., 2018). Third, it examines socioeconomic and policy challenges, such as high upfront costs, digital literacy gaps, and regulatory hurdles (Pretty et al., 2018). For example, GMO restrictions in the EU and limited IoT infrastructure in Africa slow technology diffusion (Qaim, 2020). Fourth, the review explores emerging innovations, such as blockchain for supply-chain transparency and AI-driven climate adaptation models (Kamilaris et al., 2017). Finally, it provides policy recommendations to accelerate equitable adoption, emphasizing public-private partnerships, farmer training programs, and subsidy reforms. By bridging the gap between research and real-world implementation, this review seeks to inform policymakers, agribusinesses, and smallholder farmers on strategies for sustainable agricultural transformation. Keeping all the objectives in view, this review addresses a central question: How can modern agricultural technologies contribute to sustainable intensification by enhancing productivity and land use efficiency, while overcoming adoption barriers across different socio-economic and environmental contexts? By framing the review around this question, we aim to provide a comprehensive evaluation of the role of innovations such as precision agriculture, biotechnology, smart irrigation, automation, vertical farming, and artificial intelligence in addressing global food security and environmental sustainability. This question will help unify the review and clarify its contribution to the field.

## 2 Methodological approach for the study

**Figure 1:**
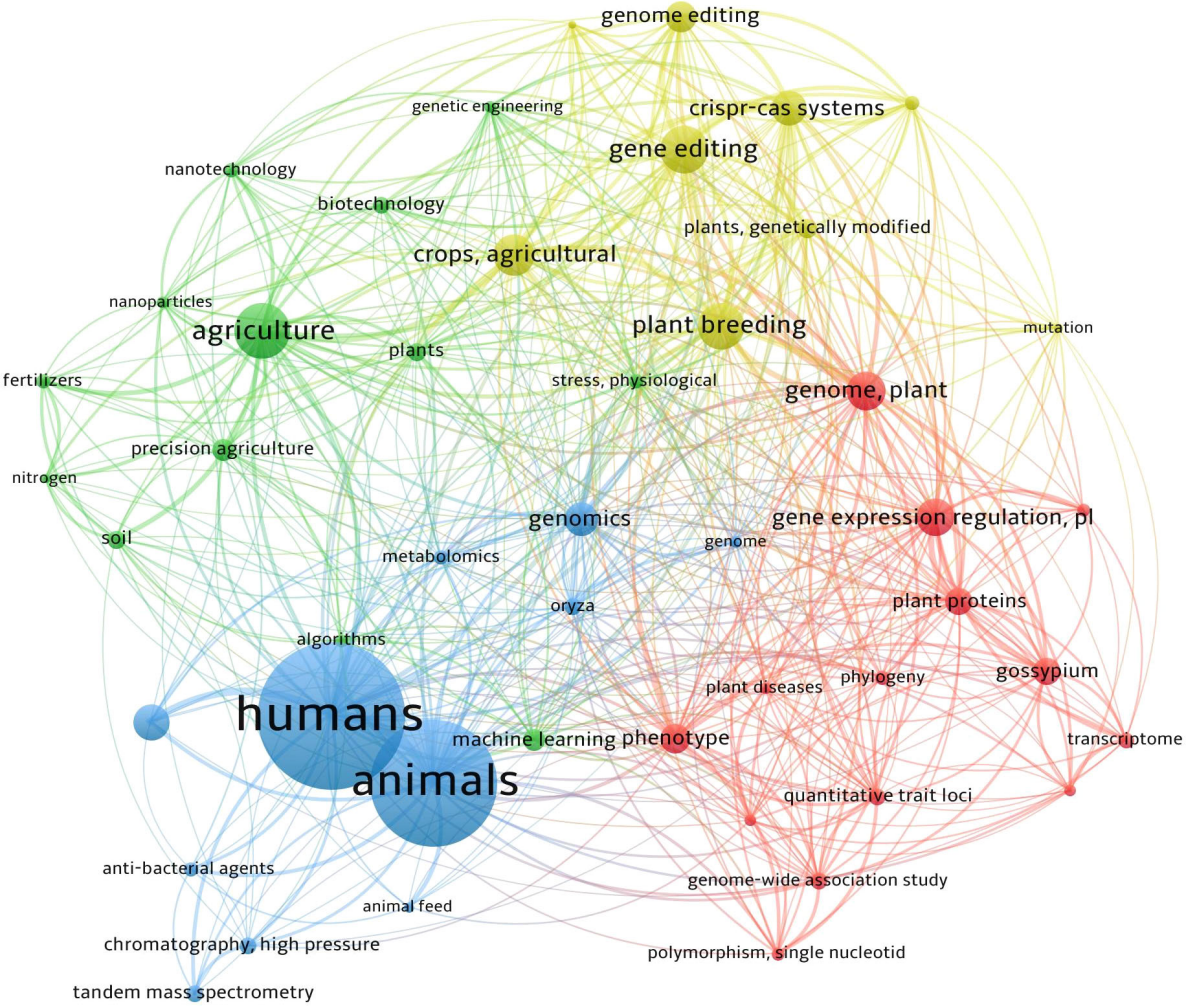
Co-occurrence network on the basis of searched articles using the relevant keywords with four wide-clusters searched on Web of Science, PubMed, google scholar, Agricola, SCOPUS, and ScienceDirect. Each node in the graph represents a keyword, and the size of the node indicates the frequency with which the keyword appears in the literature. The lines connecting the nodes represent co-occurrences of keywords; the more lines or the thicker they are, the higher the frequency of co-occurrence. The figure was prepared by VOSviewer software.

To ensure a comprehensive and systematic review of the role of modern agricultural technologies in improving agricultural productivity and land use efficiency, a structured approach was employed to select and synthesize relevant literature. This methodology section details the databases used, the keywords applied, and the inclusion and exclusion criteria to ensure transparency and reproducibility. The literature review was conducted using a combination of academic databases and grey literature sources to ensure a broad and inclusive scope. The primary databases included Web of Science, Scopus, Google Scholar, PubMed, Agricola, and ScienceDirect. Grey literature sources, such as reports from international organizations (e.g., FAO, IPCC, World Bank) and white papers from industry leaders, were also included to capture the latest developments and practical applications. A set of predefined keywords and search terms were used to identify relevant articles and reports. The primary keywords included precision agriculture, biotechnology, smart irrigation, vertical farming, automation, artificial intelligence, agricultural productivity, land use efficiency, sustainable intensification, and food security, and a VOSviewer cluster co-occurrence was drawn as shown in (**Figure 1**). These keywords were combined using Boolean operators (AND, OR) to refine the search results. For example, “precision agriculture AND agricultural productivity” or “biotechnology AND land use efficiency”. To maintain the focus and relevance of the review, specific inclusion and exclusion criteria were applied. Articles were included if they were peer-reviewed and published within the last 10 years (2015-2025). Reports and white papers from reputable organizations were also included. Studies were included if they provided empirical evidence or case studies on the impact of modern agricultural technologies on productivity and land use efficiency. Articles that discussed barriers to adoption and potential solutions were also included. Exclusion criteria included articles not related to agricultural technologies or their impact on productivity and land use efficiency. Studies focusing solely on traditional farming methods without addressing modern technologies were excluded. Non-peer-reviewed articles and opinion pieces without empirical data were also excluded. The quality of the selected studies was assessed based on methodological rigor, relevance, and transparency. Studies with robust methodologies, including randomized controlled trials, meta-analyses, and case studies with clear methodologies, were prioritized. Studies providing clear descriptions of methods, data sources, and results were also included. By adhering to these methodological standards, this review aims to provide a transparent, reproducible, and comprehensive assessment of the role of modern agricultural technologies in enhancing agricultural productivity and land use efficiency.

## 3 Overview of modern agricultural technologies

### 3.1 Precision agriculture

**Figure 2:**
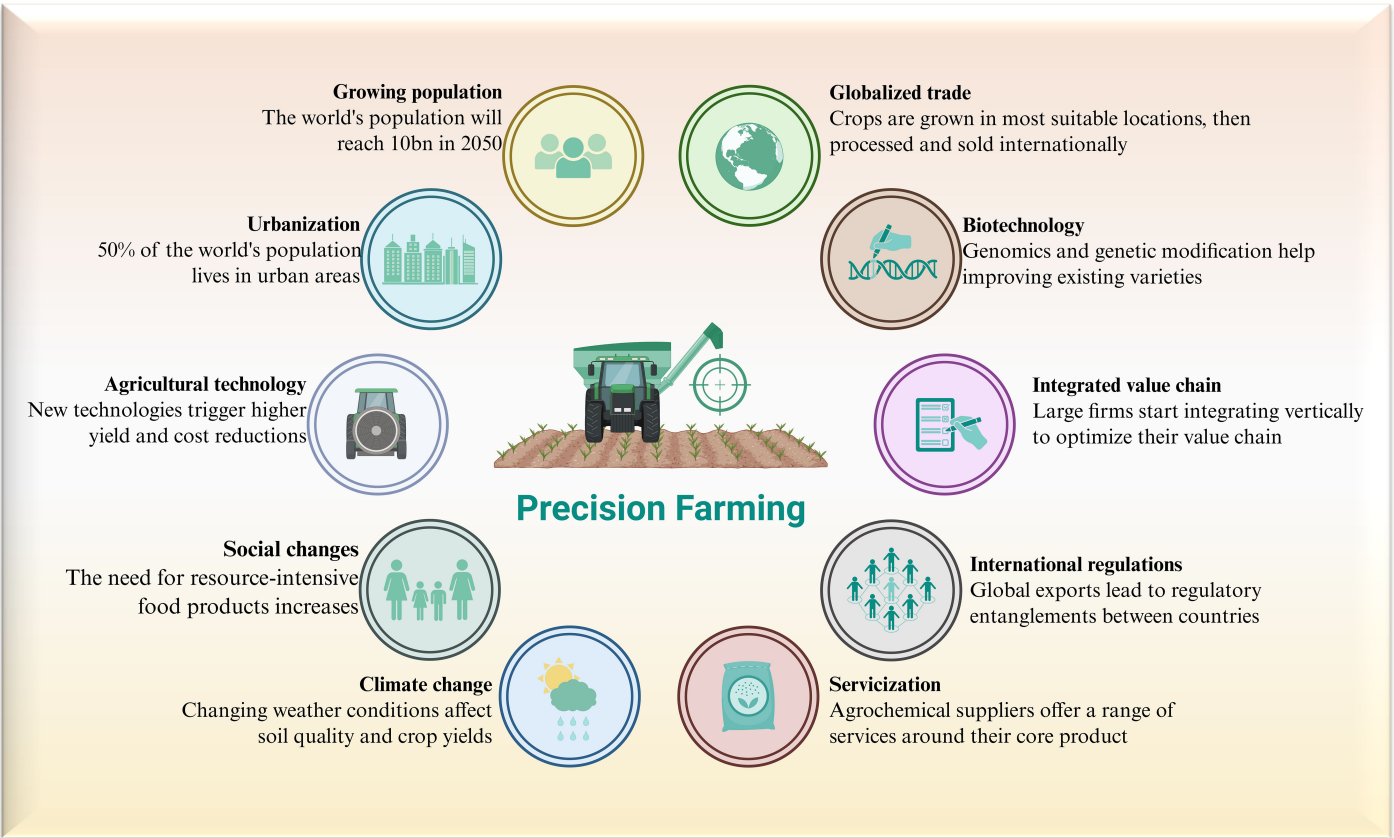
Precession agriculture as one of the modern agricultural technologies. A combined source of Biorender (www.biorender.com) and PowerPoint was used to prepare this figure.

Precision agriculture (PA) represents a transformative approach to modern farming, leveraging advanced technologies such as GPS-guided machinery, drones, and remote sensing to optimize agricultural practices (**Table 1**) and (**Figure 2**). At its core, PA relies on geospatial data to enable site-specific crop management, ensuring that inputs like water, fertilizers, and pesticides are applied precisely where needed (Gebbers and Adamchuk, 2010). GPS-guided tractors and harvesters, for instance, use real-time kinematic (RTK) positioning to achieve centimeter-level accuracy, reducing overlaps and gaps during planting and spraying (Zhang et al., 2022). This not only cuts input costs by 20-30% but also minimizes environmental impact by preventing chemical runoff into nearby ecosystems (Lowenberg-DeBoer et al., 2020). Additionally, auto-steer systems in machinery reduce operator fatigue and improve field efficiency, allowing farmers to cover larger areas with greater consistency (Pierce & Nowak, 1999). The integration of IoT-enabled sensors further enhances these systems by providing live feedback on soil moisture, nutrient levels, and crop health, enabling dynamic adjustments during operations (Boursianis et al., 2022).

**Table 1:** Modern agriculture technologies use in different field crops and their subsequent increase in yield.

Technology	Crop/Application	Yield improvement	Input reduction	Case study region
Precision Agriculture (VRT)	Corn	about 22%	Fertilizer: 15%	US Midwest
Precision Agriculture	Soybeans	about 18%	Water: 20-30%	US Midwest
Bt Cotton (GMO)	Cotton	about 31%	Pesticides: 50%	India
Drought-Tolerant Maize (WEMA)	Maize	35-50% (drought)	Water: 30%	Kenya/Tanzania
Vertical Farming (Hydroponics)	Lettuce	10-20× (per m²)	Land: 95%; Water: 95%	Singapore
AI Pest Prediction	Cotton	40% (loss reduction)	Pesticides: 30-40%	India (Maharashtra)
CRISPR-Edited Rice	Rice (C4 Project)	50% (potential)	Fertilizer: 20%	Global Trials
Automated Harvesting	Strawberries	95% efficiency	Labor: 40%	Global
Smart Irrigation (Drip)	Rice-Wheat Systems	Yield maintained	Water: 35%	India (Punjab)
Agrivoltaics	Mixed Crops	40% land efficiency	Energy: 30%	Global Trials
Solar-Powered Irrigation	Mixed Crops	300%	Diesel: 100%	Sub-Saharan Africa

Drones (UAVs) and remote sensing technologies have revolutionized precision agriculture by providing high-resolution, real-time aerial data for crop monitoring and management. Multispectral and hyperspectral cameras mounted on drones capture critical information about plant health, moisture stress, and pest infestations that are invisible to the naked eye (Zhang et al., 2022). For example, normalized difference vegetation index (NDVI) maps, derived from drone imagery, help farmers identify underperforming crop zones, allowing for targeted interventions (Mulla, 2013). Drones also enable variable-rate application (VRA) of agrochemicals, where pesticides or fertilizers are dispensed only in areas showing signs of deficiency or disease, reducing waste by up to 50% (Shafi et al., 2019). Beyond drones, satellite remote sensing offers broader-scale insights, tracking large-field crop progress, drought conditions, and even predicting yields using AI-driven analytics (Lobell et al., 2015). Companies like Planet Labs and Sentinel-2 provide frequent, high-resolution satellite imagery, empowering farmers with actionable insights at regional and global scales (Weiss et al., 2020).

The future of precision agriculture lies in the integration of these technologies into cohesive, data-driven farming systems. Advances in machine learning and AI are enabling predictive analytics that forecast disease outbreaks, optimize irrigation schedules, and even recommend hybrid seed varieties tailored to specific soil conditions (Kamilaris et al., 2017). For instance, John Deere’s Operations Center aggregates data from GPS-guided equipment, drones, and soil sensors to generate prescriptive planting maps, boosting yields by 5-10% (Schimmelpfennig, 2016). However, challenges remain, particularly for smallholder farmers, including high initial costs, technical skill requirements, and limited rural connectivity (Pretty et al., 2018). Governments and agribusinesses are addressing these barriers through subsidies, training programs, and public-private partnerships to democratize access to PA technologies. As these tools become more affordable and user-friendly, precision agriculture is poised to play a pivotal role in achieving sustainable intensification, ensuring food security while preserving natural resources for future generations.

### 3.2 Biotechnology & genetic engineering

**Figure 3:**
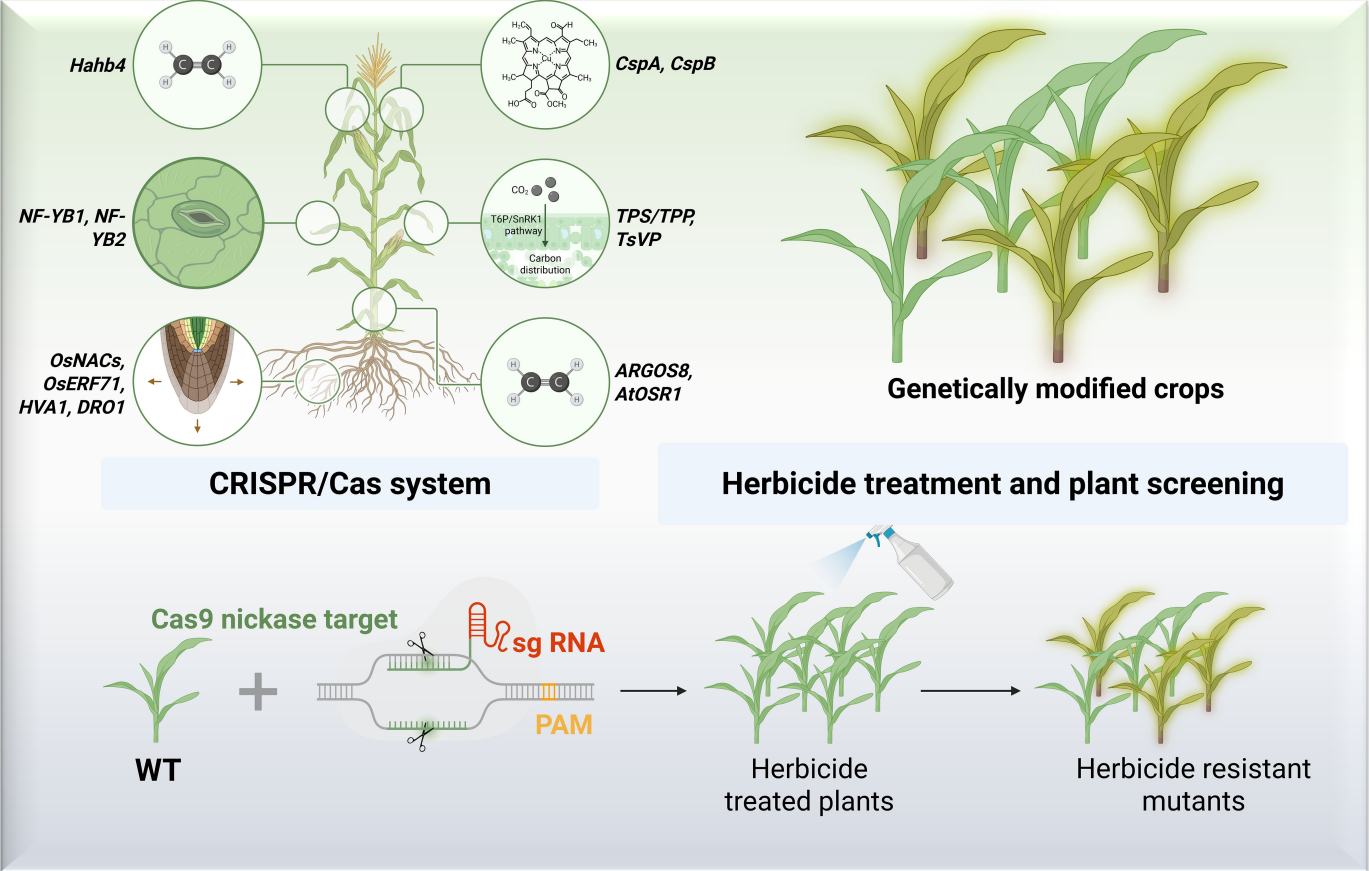
Techniques involved in the biotechnological and genetic engineering for the efficient agriculture production. Biorender application and PowerPoint was used in this figure preparation.

Recent advances in biotechnology and genetic engineering have revolutionized agriculture by developing drought-resistant crops, CRISPR-edited varieties, and genetically modified organisms (GMOs), each playing a critical role in enhancing food security under climate change (**Figure 3**). Drought-resistant crops, such as Water Efficient Maize for Africa (WEMA) and drought-tolerant wheat, are engineered to thrive in water-scarce conditions by modulating genes like *OsNAC9* in rice and *ZmPYL* in maize, improving water-use efficiency by up to 30% (Varshney et al., 2021). Similarly, CRISPR-Cas9 gene editing has enabled precise modifications in crops like soybeans and tomatoes, enhancing traits such as salt tolerance, disease resistance, and yield potential without introducing foreign DNA (Zafar et al., 2020). For example, CRISPR-edited mushrooms with non-browning traits and wheat with reduced gluten content demonstrate the technology’s versatility in addressing both agricultural and nutritional challenges (Waltz, 2016). Meanwhile, GMOs, such as Bt cotton and Golden Rice, continue to dominate markets, with Bt cotton reducing pesticide use by 50% in India (Kathage & Qaim, 2012) and Golden Rice combating vitamin A deficiency in Southeast Asia (Tang et al., 2009). Beyond single-trait modifications, stacked-trait GMOs, like drought-resistant + pest-resistant maize, are emerging, offering multi-layered resilience (Ray et al., 2020). These innovations are supported by high-throughput phenotyping and genomic selection, accelerating the breeding cycle from years to months (Crossa et al., 2017). However, regulatory hurdles and public skepticism, particularly in the EU, limit widespread adoption, underscoring the need for science-based policymaking and public education.

The functional benefits of these biotechnologies extend beyond yield enhancement to sustainability and climate adaptation. For instance, CRISPR-edited rice varieties with improved photosynthetic efficiency (C4 rice project) could boost yields by 50% while reducing water and fertilizer inputs (Ermakova et al., 2021). Similarly, gene-edited nitrogen-fixing cereals aim to reduce synthetic fertilizer dependency, mitigating nitrogen pollution (Geddes et al., 2019). In livestock, CRISPR is being used to develop disease-resistant pigs and heat-tolerant cattle, addressing challenges like African swine fever and heat stress (Tait-Burkard et al., 2018). Meanwhile, RNA interference (RNAi) technologies are creating virus-resistant crops, such as papaya ringspot virus-resistant papaya, which has saved Hawaii’s papaya industry (Tripathi et al., 2020). Synthetic biology is also contributing, with microbial consortia engineered to enhance soil health and crop resilience (Lawson et al., 2019). Despite these advances, equitable access remains a challenge, as smallholder farmers in developing regions often lack access to patented technologies (Toenniessen et al., 2008). Initiatives like the OpenCRISPR project aim to democratize gene-editing tools (Cohen, 2023), while public-sector partnerships (e.g., CIMMYT’s drought-tolerant maize) ensure affordability. As climate change intensifies, these biotechnologies will be indispensable for achieving sustainable intensification, but their success hinges on global collaboration, ethical governance, and inclusive innovation.

### 3.3 Smart irrigation & water management

Water scarcity poses one of the most significant challenges to global agriculture, with 70% of freshwater withdrawals allocated to irrigation, often inefficiently (FAO, 2020). Smart irrigation systems, integrating IoT-based sensors and drip irrigation technologies, are transforming water management by optimizing usage, reducing waste, and enhancing crop yields. Soil moisture sensors, such as capacitive probes and tensiometers, provide real-time data on water availability at varying root depths, enabling precise irrigation scheduling. These sensors transmit data via LoRaWAN or cellular networks to cloud platforms, where AI algorithms analyze trends and automate irrigation decisions (Goap et al., 2018). For example, FarmBeats by Microsoft leverages IoT and drone imagery to generate soil moisture maps, reducing water use by 20-30% in pilot farms (Agarwal et al., 2020). Complementing sensor networks, automated drip irrigation systems deliver water directly to plant roots through pressurized tubing, minimizing evaporation and runoff (Bwambale et al., 2022). Advanced systems, like Netafim’s Precision Irrigation, integrate weather forecasts and evapotranspiration (ET) data to adjust drip rates dynamically, improving water-use efficiency (WUE) by 40-60% compared to flood irrigation (Jägermeyr et al., 2021). Such innovations are critical in arid regions like Israel and California, where drip irrigation has enabled agriculture despite chronic water shortages. However, challenges persist, including high upfront costs, sensor calibration complexities, and energy demands for IoT infrastructure, particularly in developing regions.

Beyond efficiency gains, smart water management systems contribute to sustainability and climate resilience. Satellite-coupled IoT networks, such as NASA’s OpenET platform, combine remote sensing with ground sensors to monitor field-scale water consumption, aiding policymakers in drought response (Melton et al., 2022). In India’s Punjab region, IoT-enabled drip systems have reduced groundwater depletion in rice-wheat systems by 35% while maintaining yields (Singh et al., 2021). Emerging technologies like electrochemical sensors detect nutrient leaching in real time, allowing farmers to adjust fertigation schedules, thus preventing pollution. Solar-powered IoT systems are also gaining traction, addressing energy barriers in off-grid areas; for instance, SunCulture’s solar drip kits in Kenya have cut water and energy costs by 50% for smallholders (Burney et al., 2023). Meanwhile, blockchain-based water trading platforms, piloted in Australia’s Murray-Darling Basin, enable transparent allocation of saved water resources (Wheeler, 2022). Despite these advances, scaling smart irrigation requires policy support (e.g., subsidies for drip systems), farmer training, and interoperable IoT standards (Rose et al., 2021).

### 3.4 Automation & robotics technologies

**Figure 4:**
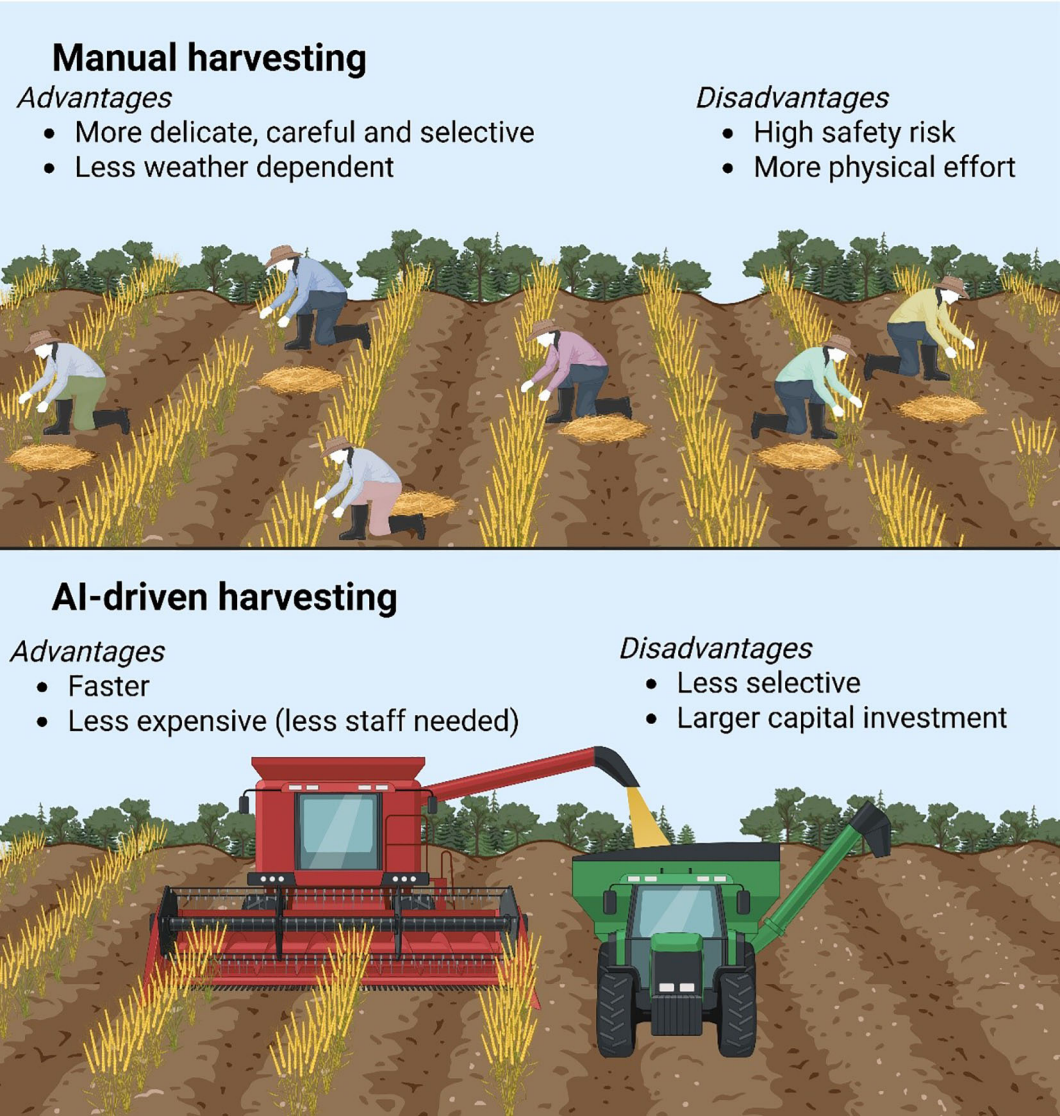
Automation and AI-driven harvesting technologies and their advantages and disadvantages. This figure was prepared through Biorender application sources (www.biorender.com).

The integration of autonomous tractors and AI-driven harvesting systems is revolutionizing agricultural productivity by addressing labor shortages, reducing operational costs, and enhancing precision in field operations (**Figure 4**). Self-driving tractors, such as John Deere’s 8R Autonomous and Case IH’s Autonomous Concept Vehicle, utilize GPS, LiDAR, and computer vision to navigate fields with centimeter-level accuracy, enabling 24/7 operation without human intervention (Lowenberg-DeBoer et al., 2020). These systems rely on real-time kinematic (RTK) positioning and machine learning algorithms to avoid obstacles, optimize path planning, and adjust for terrain variations, reducing fuel consumption by 15-20% compared to conventional tractors. Complementing autonomous tractors, robotic implements, like autonomous planters and sprayers, leverage IoT connectivity to apply seeds, fertilizers, and pesticides at variable rates based on soil and crop data, minimizing input waste by 30-50% (Zhang et al., 2022). For example, Blue River Technology’s “See & Spray” robot uses computer vision and AI to distinguish crops from weeds, targeting herbicide sprays with 95% accuracy. Similarly, harvesting robots, such as Agrobot’s strawberry picker and FFRobotics’ apple harvester, employ 3D cameras, robotic arms, and AI classifiers to identify ripe produce and harvest it without damage, addressing the $30 billion annual global labor gap in fruit and vegetable production (Bac et al., 2021). Despite these advancements, challenges like high capital costs ($500,000+ per autonomous tractor) and regulatory hurdles for field safety limit widespread adoption, particularly for small-scale farmers (Schimmelpfennig, 2022).

The future of agricultural robotics lies in swarm robotics, edge AI, and human-robot collaboration, which promise to further optimize efficiency and scalability. Swarm robotics, where multiple small robots (e.g., FarmWise’s weeding robots) work collaboratively, can cover large fields faster while reducing soil compaction. Edge AI, deployed on devices like NVIDIA’s Jetson-powered harvesters, processes data locally to make real-time decisions without cloud dependency, critical for remote farms with limited connectivity (Kamilaris et al., 2023). Meanwhile, human-robot collaboration systems, such as Sweeper’s greenhouse pepper harvester, allow human workers to oversee fleets of robots, improving productivity by 40%. AI-driven predictive maintenance is another breakthrough, using sensor data and neural networks to preemptively service machinery, reducing downtime by 25%. Economic analyses suggest that full automation could reduce global farming costs by $50 billion annually by 2030 (World Economic Forum, 2023), but equitable access requires subscription-based robotics-as-a-service (RaaS) models, as piloted by Monarch Tractor’s pay-per-use program (Gonzalez-de-Santos et al., 2020). Policy initiatives, such as the EU’s Agricultural Robotics Act (2024), aim to standardize safety protocols and subsidies to accelerate adoption (European Commission, 2023). As these technologies mature, autonomous systems will be pivotal in meeting the 70% increase in food demand by 2050, while adhering to sustainable practices (FAO, 2023).

### 3.5 Vertical & urban farming

**Figure 5:**
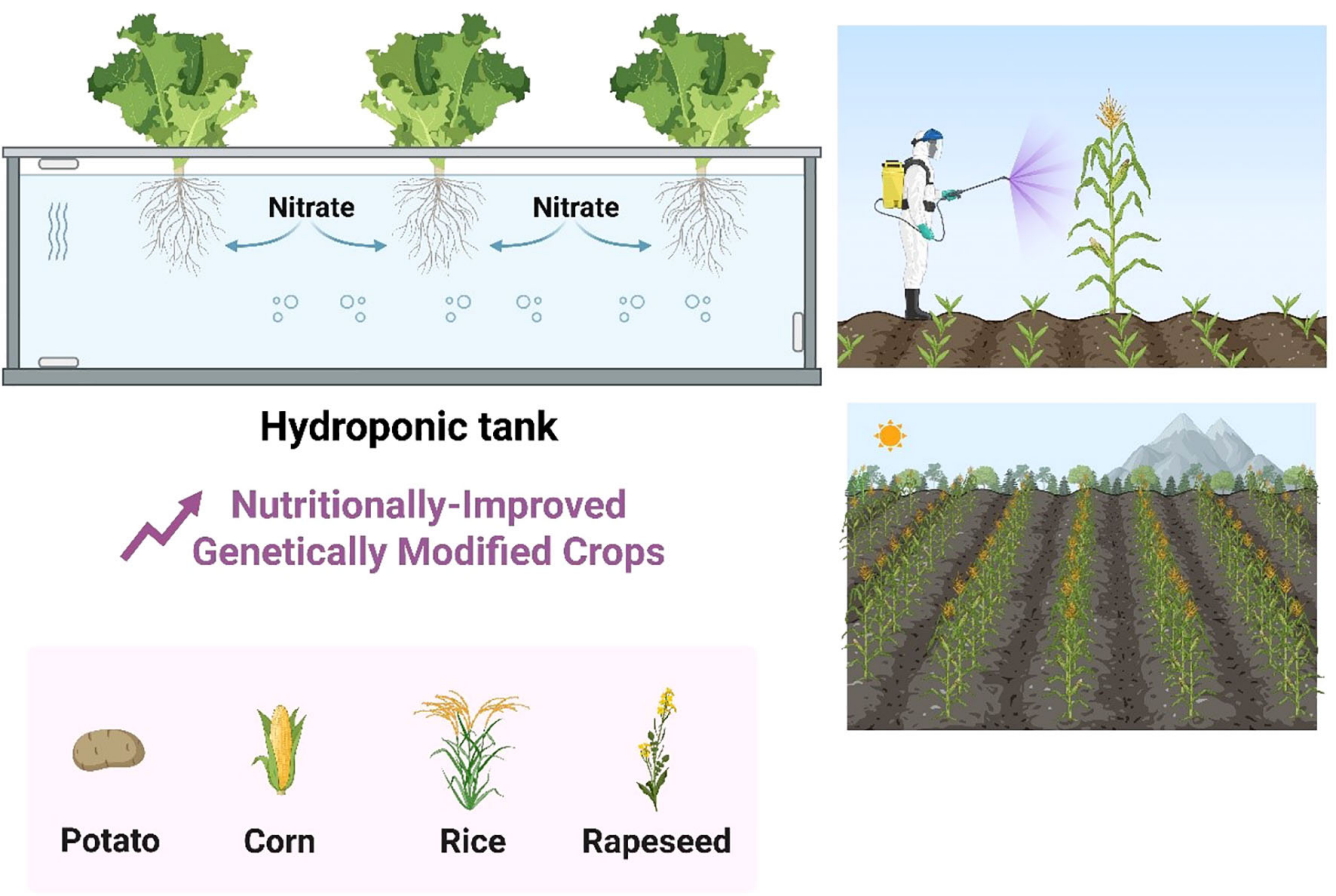
Improved crop production in agriculture via novel technologies i.e., hydroponics and genetically modified crops. The source used for this figure was Biorender and PowerPoint.

Vertical and urban farming systems, including hydroponics, aeroponics, and controlled-environment agriculture (CEA), are redefining agricultural productivity by enabling year-round crop production in urban areas with minimal land and water use. Hydroponic systems, which grow plants in nutrient-rich water solutions without soil, can achieve 10–12 times higher yields per square meter compared to traditional farming while using 90% less water (Barbosa et al., 2023). Advanced hydroponic configurations, such as nutrient film technique (NFT) and deep-water culture (DWC), optimize root oxygenation and nutrient delivery, making them ideal for leafy greens like lettuce and herbs (Lei and Engeseth, 2021). Aeroponics, a more resource-efficient variant, suspends plant roots in air and intermittently mists them with nutrient solutions, reducing water usage by 95% and accelerating growth rates by 30% (Lakhiar et al., 2018). NASA’s adoption of aeroponics for space missions underscores its potential for extreme-environment farming (Monje et al., 2003). These soilless systems are often integrated into vertical farms, multi-tiered indoor facilities that maximize space efficiency in urban settings e.g., hydroponics as shown in (**Figure 5**). For instance, AeroFarms’ 9-story vertical farm in New Jersey produces 2 million pounds of greens annually on just 1% of the land required by conventional agriculture (Banerjee and Adenaeuer, 2021). The success of vertical farming hinges on controlled-environment agriculture (CEA), which uses LED lighting, climate control, and AI-driven monitoring to replicate optimal growing conditions. Dynamic LED spectra, tailored to crop-specific photosynthetic needs, can enhance yields by 40% while reducing energy costs by 25% (Kozai and Niu, 2016). For example, Japan’s Spread Co. uses pink LEDs (a blend of red and blue wavelengths) to boost lettuce growth (Yanagi et al., 1996). Meanwhile, AI-powered climate control systems, like Intelligent Growth Solutions’ (IGS) Growth Towers, adjust temperature, humidity, and CO_2_ levels in real time based on plant telemetry (Beacham et al., 2023). Automated nutrient dosing and predictive analytics further minimize waste; Gotham Greens’ rooftop farms employ IoT sensors to track 50+ variables daily, achieving 99% nutrient-use efficiency (Carotti et al., 2021). Despite these advantages, high energy demands (up to 3,500 kWh per ton of produce) and capital costs ($100–$300 per square foot) remain barriers (Avgoustaki and Xydis, 2022). Innovations like solar-powered vertical farms (e.g., Sundrop Farms’ Australia facility) and modular, container-based systems (e.g., Freight Farms’ Leafy Green Machine) aim to improve affordability and scalability.

Urban farming also addresses critical socioeconomic and environmental challenges, including food deserts, supply chain resilience, and carbon footprints. By locating farms within cities, transportation emissions for leafy greens can be reduced by 90% (Lam et al., 2021). Projects like Singapore’s Sky Greens and Berlin’s Infarm demonstrate how vertical farms can supply 10–15% of a city’s fresh produce (Sanyé-Mengual et al., 2022). Policy support, such as Singapore’s 30×30 Initiative (30% local food production by 2030) and New York’s Urban Agriculture Tax Credit, is accelerating adoption (Cohen, 2023). Future advancements may include bio-integrated systems, where microbial inoculants enhance nutrient uptake (Al-Kodmany, 2024), and circular-economy models, such as using food waste-derived fertilizers (Ellen MacArthur Foundation, 2023). As urbanization intensifies, vertical and urban farming will be pivotal in achieving SDG 11 (Sustainable Cities) and SDG 12 (Responsible Consumption), ensuring food security, and food quality while mitigating agriculture’s environmental impact (**Figure 5**).

### 3.6 Big data & AI in agriculture

The integration of Big Data and Artificial Intelligence (AI) into agriculture has ushered in a new era of precision farming, enabling farmers to make data-driven decisions that optimize productivity, reduce waste, and enhance sustainability. Predictive analytics, powered by machine learning (ML) algorithms, leverages historical and real-time data, such as weather patterns, soil conditions, and crop health, to forecast outcomes like yield potential, pest outbreaks, and irrigation needs with over 90% accuracy (Kamilaris et al., 2023). For example, IBM’s Watson Decision Platform for Agriculture combines satellite imagery, IoT sensor data, and weather forecasts to predict crop stress up to 14 days in advance, allowing preemptive interventions (Zhang et al., 2023). Similarly, The Climate Corporation’s FieldView uses AI to analyze field-specific data and generate prescriptive planting maps, which have been shown to increase corn yields by 5–10 bushels per acre (Schimmelpfennig and Chermak, 2022). These systems rely on neural networks and ensemble modeling to process vast datasets, including multispectral drone imagery (for detecting nutrient deficiencies) and soil moisture sensors (for optimizing irrigation schedules) (Lacerda et al., 2022). However, the effectiveness of predictive models depends on data quality and interoperability, with challenges like fragmented farm records and proprietary software limitations hindering seamless integration (Wolfert et al., 2022).

Farm management software (FMS) platforms, such as Granular, Agworld, and FarmLogs, serve as centralized hubs for data aggregation, analysis, and decision support, streamlining operations from planting to harvest. These tools enable variable-rate application (VRA) of inputs by integrating GPS-guided machinery data with soil and yield maps, reducing fertilizer use by 20–30% while maintaining yields (Lowenberg-DeBoer et al., 2023). Advanced FMS solutions also incorporate blockchain technology for supply chain transparency, allowing consumers to trace produce from farm to table, a feature piloted by Walmart’s IBM Food Trust for leafy greens (DeFranco et al., 2023). AI-driven robotics, such as Blue River Technology’s See & Spray, further enhance FMS by autonomously targeting weeds with herbicides, cutting chemical usage by 90%. Despite these benefits, adoption barriers persist, particularly among smallholder farmers, due to high subscription costs (e.g., $500–$2,000 annually for premium FMS) and limited digital literacy. To address this, initiatives like FAO’s Digital Villages Program provide subsidized training and low-bandwidth FMS tools for rural communities (FAO, 2023). Looking ahead, edge computing, where AI processes data locally on devices like John Deere’s embedded farm computers, will reduce reliance on cloud connectivity, a critical advancement for remote areas.

The future of Big Data and AI in agriculture lies in autonomous decision-making systems that merge predictive analytics with robotic execution. For instance, Taranis’ AI-powered scouting drones autonomously detect diseases like soybean rust and trigger targeted sprays without human intervention (Navrozidis et al., 2023). Meanwhile, generative AI models, such as ChatGPT for Agriculture, are being trained to provide real-time agronomic advice by synthesizing research papers, extension reports, and field data. Digital twin technology, which creates virtual replicas of farms, enables simulation-based optimization of crop rotations and resource allocation (Bauer et al., 2023). Policy frameworks, like the EU’s Digital Farming Act (2024), aim to standardize data sharing and ethics in AI agriculture (European Commission, 2023). As these technologies mature, they will be pivotal in meeting the 60% increase in global food demand by 2050, while reducing agriculture’s carbon footprint by 20% through precision resource use (World Bank, 2023).

## 4 Impact on agricultural productivity

Numerous modern technologies, including Vertical Farming (CEA), Drip Irrigation, Precision Fertilization, Autonomous Tractors CRISPR Nitrogen-Fixing Crops, Solar-Powered IoT Sensors, AI-Driven Weed Control, Blockchain Supply Chains, Swarm Robotics, Recycled Hydroponic Water have been used to improve the agricultural productivity as shown in **Table 2**. Following are the case studies showing an improvement in yield through the application of these modern technologies.

**Table 2:** Modern technologies and their impact on improved agriculture efficiency and environmental benefits.

Technology	Resource saved	Efficiency gain	Environmental benefit	Key example
Vertical Farming (CEA)	Land	95-98% reduction	Prevents deforestation	AeroFarms, USA
Drip Irrigation	Water	40-60% reduction	Reduces groundwater depletion	Netafim, Israel
Precision Fertilization	Fertilizer	20-30% reduction	Lowers nitrogen pollution	US Corn Belt
Autonomous Tractors	Fuel	15-20% reduction	Cuts CO_2_ emissions	John Deere 8R
CRISPR Nitrogen-Fixing Crops	Synthetic Fertilizer	Potential 50% reduction	Mitigates N_2_O emissions	Geddes et al. (2019)
Solar-Powered IoT Sensors	Energy	100% renewable	Eliminates diesel pumps	SunCulture, Kenya
AI-Driven Weed Control	Herbicides	90% reduction	Protects soil microbiomes	Blue River Technology
Blockchain Supply Chains	Food Waste	Recall time: 2.2s → 7d	Reduces landfill emissions	Walmart IBM Food Trust
Swarm Robotics	Soil Compaction	50% reduction	Preserves soil structure	FarmWise
Recycled Hydroponic Water	Water	95% reuse rate	Conserves freshwater	Gotham Greens

### 4.1 Case studies showing yield improvements

Modern agricultural technologies have demonstrated measurable success in boosting crop yields across various farming systems. One notable example comes from precision farming applications in the US Midwest, where GPS-guided variable-rate technology (VRT) has transformed corn and soybean production. Research conducted by the USDA Agricultural Research Service (2023) across Iowa, Illinois, and Nebraska farms revealed that precision nitrogen management systems increased corn yields by 22% while simultaneously reducing fertilizer application by 15%. These systems utilize soil sensors and satellite imagery to create customized nutrient application maps, ensuring optimal fertilization while minimizing environmental runoff. Similarly, soybean operations employing auto-steer planters and yield monitoring technology achieved 18% higher yields through improved seed spacing uniformity and depth control (Lowenberg-DeBoer et al., 2023). The economic impact has been significant, with participating farms reporting $150–$200 per acre in additional net returns.

In drought-prone regions of Sub-Saharan Africa, biotechnology has played a crucial role in stabilizing yields. The Water Efficient Maize for Africa (WEMA) project, deploying genetically modified drought-tolerant hybrids, has demonstrated remarkable results. Field trials across Kenya and Tanzania showed these varieties maintained 35–50% higher yields during drought conditions compared to conventional maize, with no yield penalty in normal rainfall years. The incorporation of genes like ZmPYL, which regulate plant water-use efficiency, has enabled deeper root development and reduced moisture stress. Smallholder farmers adopting these varieties reported 25% higher incomes due to reduced crop failure rates, highlighting the technology’s potential for climate resilience. These gains are particularly impactful in regions where maize serves as both a staple food and primary income source for millions of households. Artificial intelligence has emerged as a powerful tool for protecting yields from pest pressures. In India’s cotton-growing regions of Maharashtra, IBM’s AI-powered pest prediction system has helped farmers combat devastating pink bollworm outbreaks. By analyzing historical pest data, weather patterns, and field scouting reports from drones, the system provides early warnings that enable targeted pesticide applications. Pilot implementations demonstrated a reduction in crop losses from 60% to 20%, effectively rescuing 40% of potential yield (IBM Research, 2023). The technology’s predictive accuracy continues to improve through machine learning algorithms that incorporate real-time field data from IoT sensors. This approach not only preserves yields but also reduces unnecessary pesticide use by 30–40%, creating both economic and environmental benefits.

Controlled environment agriculture has redefined productivity benchmarks for leafy greens and herbs. Singapore’s Sky Greens vertical farm exemplifies this potential, achieving annual lettuce yields of 1,500 kg per square meter compared to 80 kg in traditional soil-based systems (Sanyé-Mengual et al., 2023). The hydroponic operation combines multi-tiered growing systems with spectrally optimized LED lighting to enable year-round production cycles unaffected by external weather conditions. Remarkably, this productivity is achieved using 95% less water than field production through closed-loop irrigation systems. Such innovations are transforming urban food security, with a single vertical farm now capable of supplying 5–10% of a city’s fresh vegetable demand while using minimal land area. These case studies collectively demonstrate how targeted technological applications can address distinct productivity challenges across diverse agricultural contexts.

### 4.2 Role of genetically modified crops in pest/disease resistance

Genetically modified (GM) crops have revolutionized modern agriculture by providing robust solutions to pest and disease pressures that traditionally caused significant yield losses. Through targeted genetic engineering, scientists have developed crop varieties with built-in resistance mechanisms that reduce reliance on chemical pesticides while improving farm productivity and sustainability. The most prominent example remains Bt (Bacillus thuringiensis) crops, which now dominate global production of several staple crops. Bt cotton, engineered to produce insecticidal proteins toxic to bollworms and other lepidopteran pests, has demonstrated remarkable success across multiple continents. In India, the world’s largest cotton producer, Bt varieties reduced pesticide applications by 50% while increasing yields by 31% and farmer profits by 88% according to a meta-analysis of farm surveys (Kathage & Qaim, 2012). Similar results were observed in China, where Bt cotton adoption decreased pesticide use by 65,000 metric tons annually while reducing farmer pesticide poisoning incidents by 50-70% (Wu et al., 2008).

Beyond insect resistance, genetic engineering has produced crops with enhanced viral and bacterial disease resistance. The Rainbow papaya, developed to resist the devastating papaya ringspot virus (PRSV), saved Hawaii’s papaya industry from collapse in the 1990s. Field trials showed 100% of conventional papaya trees became infected within 11 months, while 100% of GM trees remained healthy (Gonsalves, 2006). Similarly, researchers have developed GM potatoes resistant to late blight (Phytophthora infestans), the pathogen responsible for the Irish Potato Famine, which can reduce yields by 80% in untreated fields. Field trials in the U.S. and Europe demonstrated these GM varieties required 80-90% fewer fungicide applications while maintaining yield parity with heavily sprayed conventional potatoes (Haverkort et al., 2016). The emergence of CRISPR-Cas9 gene editing has accelerated development of disease-resistant crops by enabling precise modifications to plant immune systems. Scientists have used CRISPR to knock out susceptibility genes in wheat to create powdery mildew-resistant varieties (Wang et al., 2014), and to enhance resistance to bacterial blight in rice (Zhou et al., 2015). Unlike traditional GM approaches that introduce foreign DNA, many CRISPR-edited crops contain only minor, targeted changes to existing plant genomes, potentially easing regulatory hurdles and public acceptance challenges. The economic and environmental benefits of pest- and disease-resistant GM crops are substantial. A global meta-analysis estimated that GM crop adoption reduced pesticide use by 37%, increased crop yields by 22%, and increased farmer profits by 68% compared to conventional crops (Klümper & Qaim, 2014). These benefits are particularly pronounced in developing countries, where smallholder farmers often lack access to effective pest control alternatives. However, the evolution of pest resistance poses an ongoing challenge, necessitating integrated resistance management strategies such as refuge planting and gene pyramiding to preserve the long-term efficacy of these technologies.

### 4.3 Efficiency gains from automation and reduced labor dependency

The agricultural sector has witnessed transformative efficiency improvements through automation technologies that address critical labor shortages while optimizing production processes. Autonomous farming equipment, including self-driving tractors and robotic harvesters, has demonstrated remarkable productivity gains,a single autonomous tractor can operate continuously for 24 hours, achieving the work output of 2–3 traditional tractors while reducing labor costs by up to 40% (Lowenberg-DeBoer et al., 2020). These systems utilize advanced GPS guidance with centimeter-level accuracy, coupled with machine vision for obstacle detection, enabling precise field operations without human intervention. Fruit and vegetable harvesting, traditionally one of the most labor-intensive agricultural operations, has seen particularly dramatic improvements through robotic systems. The latest strawberry harvesting robots can pick a berry every 1.5 seconds with 95% accuracy, matching human picker speeds while operating continuously without breaks (Bac et al., 2021). Similarly, automated lettuce thinning machines can process 1 acre in 30 minutes, a task that would require 10–15 human laborers working an entire day (Raja et al., 2020).

The economic impact of agricultural automation extends beyond direct labor substitution. Precision automation enables optimal timing of operations, with studies showing that automated planting systems can increase yields by 5-10% simply by ensuring perfect planting dates and depth consistency (Schimmelpfennig and Chermak, 2022). Automated milking systems in dairy operations have increased milk production by 10-15% through optimized milking schedules and improved animal welfare monitoring. Perhaps most significantly, automation addresses the structural labor shortages plaguing global agriculture - the U.S. farm workforce has declined by 75% since 1950 despite a 250% increase in output (USDA-ARS, 2023), a productivity revolution made possible largely through mechanization and automation. Emerging technologies are pushing these efficiency gains further. Swarm robotics systems, where multiple small autonomous units collaborate on tasks like weeding or soil sampling, can cover fields 3–5 times faster than conventional equipment while reducing soil compaction. AI-powered quality control systems in processing facilities can sort produce at speeds of 15–20 items per second with superhuman accuracy, reducing post-harvest losses by up to 30% (Zhang et al., 2023). As these technologies mature and scale, their potential to sustainably intensify agricultural production while mitigating labor constraints will become increasingly vital to global food security.

## 5 Role in land use efficiency

### 5.1 Impact on land footprint through vertical farming & high-density cultivation

Vertical farming and high-density cultivation systems are revolutionizing land use efficiency in agriculture, offering solutions to the growing pressure on arable land resources. These innovative production methods achieve remarkable space optimization, with vertical farms producing 10–20 times more crop yield per unit area compared to conventional field agriculture (Banerjee and Adenaeuer, 2021). A single 10,000 square foot vertical farm can match the production output of 50–100 acres of traditional farmland for leafy greens and herbs, effectively reducing the agricultural land footprint by 95-98% (Sanyé-Mengual et al., 2023). This extraordinary land-use efficiency is achieved through multi-layer growing systems (typically 5–12 stacked levels), precise climate control, and year-round production cycles unaffected by seasonal limitations.

The land-saving potential of these technologies is particularly valuable in urban environments, where they enable local food production on non-arable land such as rooftops, abandoned buildings, and underground spaces. For instance, Singapore’s Sky Greens vertical farm produces 1 ton of vegetables daily on just 0.2 hectares of vertical space - a land productivity rate 10–15 times higher than conventional farms. Similarly, AeroFarms’ 70,000 square foot New Jersey facility yields 2 million pounds of greens annually using 1% of the land required by field production (AeroFarms, 2023). These systems also demonstrate superior water-use efficiency, requiring 70-95% less water than soil-based agriculture through closed-loop hydroponic and aeroponic systems that recycle water and nutrients (Barbosa et al., 2023).

Beyond space efficiency, vertical farming contributes to land conservation by:

1. Reducing agricultural expansion into natural ecosystems (potentially saving millions of hectares of forests and grasslands)
2. Enabling brownfield redevelopment (converting urban wastelands to productive use)
3. Decreasing transportation-related land use through localized production

However, these benefits must be weighed against the energy requirements of controlled environment agriculture, which currently limits these systems to high-value crops like leafy greens, herbs, and microgreens. Ongoing advances in renewable energy integration and energy-efficient LED lighting are progressively improving the sustainability profile of vertical farming systems (Avgoustaki and Xydis, 2022). As these technologies mature, their role in achieving sustainable land use efficiency while meeting urban food demand will continue to expand.

### 5.2 Sustainable intensification vs. traditional expansion

The global agricultural sector faces a critical dilemma in meeting rising food demands: whether to pursue traditional horizontal expansion (converting natural ecosystems to farmland) or adopt sustainable intensification (increasing productivity on existing agricultural land). Mounting evidence suggests sustainable intensification offers a more ecologically and economically viable path forward.

Sustainable intensification leverages technological innovations to boost yields while minimizing environmental impacts. Precision agriculture systems have demonstrated 20-30% higher yields on existing farmland through optimized input use (USDA-ARS, 2023), while advanced breeding techniques have developed crop varieties yielding 40-60% more with the same land and water inputs (Ray et al., 2020). This approach preserves natural ecosystems that provide essential services - the conversion of just 1 hectare of tropical forest to farmland releases 200–500 tons of stored carbon while destroying biodiversity habitats (West et al., 2020). Intensive vertical farming systems amplify these benefits, producing 10-20× higher yields per unit area than field agriculture while using 95% less water (Banerjee and Adenaeuer, 2021).

In contrast, traditional expansion continues driving alarming environmental degradation:

- Agriculture accounts for 80% of global deforestation (FAO, 2022)
- Each year, 10 million hectares of productive land are lost to degradation
- Soil erosion from expanded cultivation removes 24 billion tons of fertile topsoil annually (Borrelli et al., 2020)

Economic analyses reveal intensification’s superior long-term value. While land expansion provides short-term production gains, it incurs $6.3 trillion annually in hidden environmental costs from biodiversity loss, carbon emissions, and water depletion (TEEB, 2021). Sustainable intensification alternatives typically show 3-5× higher benefit-cost ratios when accounting for ecosystem services (Pretty et al., 2018).

The most promising solutions integrate multiple intensification strategies:

1. Precision resource management (sensors, VRT, smart irrigation)
2. Advanced genetics (drought-tolerant/high-yielding varieties)
3. Ecological intensification (cover crops, integrated pest management)
4. Vertical production systems (CEA, urban agriculture)

As climate change exacerbates land constraints, sustainable intensification emerges as the only viable path to achieve global food security while meeting climate commitments and preserving critical ecosystems. Policy initiatives like the EU’s Farm to Fork Strategy now actively promote this transition through incentives for precision farming and agroecological practices (European Commission, 2023).

### 5.3 Soil health monitoring and reduced degradation via smart farming

Modern agriculture faces a critical challenge in maintaining soil health, with an estimated 24 billion tons of fertile soil lost annually to erosion, nutrient depletion, and improper land management (Borrelli et al., 2020). Smart farming technologies are revolutionizing soil conservation by enabling real-time monitoring, data-driven interventions, and precision conservation techniques that mitigate degradation while improving productivity.

#### 5.3.1 Real-time soil health monitoring

Advances in sensor technology and remote sensing have transformed how farmers assess and manage soil conditions. Wireless IoT sensors embedded in fields continuously track soil moisture, temperature, pH, and nutrient levels at varying depths, transmitting data to cloud-based platforms for analysis. This granular monitoring allows for immediate adjustments in irrigation and fertilization, preventing both waterlogging and nutrient runoff. Meanwhile, satellite and drone-based hyperspectral imaging detects early signs of degradation, such as salinization or organic matter loss, before they become irreversible (Pouladi et al., 2023). AI-powered analytics further enhance decision-making by predicting soil health trends based on historical data, weather forecasts, and crop rotations, recommending optimal cover cropping, reduced tillage, and organic amendments (Bauer et al., 2023).

#### 5.3.2 Precision conservation techniques

Smart farming enables targeted conservation strategies that minimize soil disturbance while maximizing fertility. Variable-rate tillage (VRT) systems use GPS and soil maps to reduce mechanical disruption in erosion-prone zones, cutting soil loss by 30–50% compared to conventional tillage. Automated cover crop seeding systems ensure continuous ground cover during fallow periods, which has been shown to increase water retention by 15–20% and boost organic carbon levels within just 3–5 years (Hart et al., 2023). Smart irrigation systems, integrated with soil moisture sensors, prevent salinization and waterlogging by delivering precise amounts of water only where needed, reducing degradation risks by 40% (Bwambale et al., 2022).

#### 5.3.3 Economic and environmental benefits

The adoption of smart soil management practices delivers measurable benefits for both farmers and ecosystems. Farms utilizing these technologies report 10–25% higher crop yields due to optimized nutrient use and reduced stress from drought or waterlogging (Zhang et al., 2023). Additionally, precision soil management enhances carbon sequestration, with studies showing an increase of 1–2 tons of carbon per hectare annually (McClelland et al., 2022). Innovations like autonomous robotic weeders (e.g., FarmWise’s AI-powered systems) further protect soil microbiomes by minimizing herbicide use, preserving long-term fertility.

## 6 Challenges & barriers

Despite the transformative potential of smart farming and precision agriculture, several significant barriers hinder widespread adoption, particularly among smallholder farmers and in developing regions as shown in (**Table 3**). These challenges span economic, technical, regulatory, and environmental concerns, which must be addressed to ensure equitable and sustainable implementation.

**Table 3:** Barrier’s category, challenges, their impact, and potential solutions in modern agriculture technologies to improve productivity.

Barrier category	Specific challenge	Impact	Affected group	Potential solutions
Economic	High upfront costs	$500K/tractor	Smallholder farmers	Leasing models (Hello Tractor)
Technical	Rural internet gaps	50% unconnected farms	Developing regions	Low-bandwidth IoT (FAO Digital Villages)
Regulatory	GMO bans in EU	32 countries restrict	Agribusinesses	Science-based policymaking
Environmental	E-waste from sensors	53M tons/year	Global	Modular/repairable designs
Skills Gap	Digital literacy	28% trained farmers	Rural communities	Voice-based mobile apps
Energy Demands	Vertical farm electricity	3,500 kWh/ton	Urban farms	Solar integration (Sundrop Farms)
Data Ownership	Privacy conflicts	Legal disputes	Farmers vs. agtech firms	Blockchain-based data sharing
Monoculture Risks	Reduced biodiversity	Pest vulnerability	Large-scale farms	Crop rotation mandates

### 6.1 Economic barriers

The upfront investment required for advanced agricultural technologies remains prohibitive for many farmers. Autonomous tractors can cost $300,000–$500,000, while IoT-based soil monitoring systems require $500–$2,000 per hectare in sensor and connectivity infrastructure (Lowenberg-DeBoer et al., 2023). Smallholder farmers, who constitute 84% of the world’s farms (FAO, 2022), often lack access to financing for such investments. Even when technologies are affordable, rural internet connectivity gaps limit the functionality of cloud-based farm management platforms. Emerging solutions, such as pay-per-use leasing models (e.g., Hello Tractor’s shared-equipment platform) and low-cost, solar-powered sensors, are helping bridge this gap, but scalability remains a challenge (World Bank, 2023).

The adoption of advanced agricultural technologies continues to face significant economic challenges, particularly in low-income regions where smallholder farmers dominate production. High upfront costs, limited access to credit, and uncertain returns on investment hinder the uptake of innovations such as precision farming tools, automated livestock monitoring, and AI-driven pest management (Yan et al., 2025; Yue et al., 2025). For instance, while autonomous laser weeding systems (Tran et al., 2023) and precision livestock technologies (Palma-Molina et al., 2023) offer long-term efficiency gains, their initial costs remain prohibitive for resource-constrained farmers. Similarly, emerging biocontrol strategies for pests (Yue et al., 2025) and sustainable poultry farming innovations (Bist et al., 2024) require financial investments that many small-scale producers cannot afford without external support. To overcome these economic barriers, innovative financing models such as leasing and pay-per-use services are being explored. For example, Hello Tractor’s shared-equipment platform allows farmers to access autonomous tractors without the need for large upfront payments. Additionally, low-cost, solar-powered sensors are being developed to make technology more affordable and scalable in developing regions. Governments play a crucial role in reducing financial burdens through subsidies and incentives. For example, India’s agri-drone subsidy scheme (2022-2023) covers up to 100% of costs for farmer cooperatives, significantly lowering entry barriers for smallholders (Biradar and Hosalli, 2024). Similarly, Kenya’s Pay-As-You-Go (PAYG) solar irrigation initiatives, supported by the World Bank, allow farmers to adopt climate-smart technologies through flexible payment plans, making them accessible to low-income producers (Orjuela-Garzon et al., 2024). In the European Union, the Common Agricultural Policy (CAP) provides digital grants to farmers adopting smart technologies, ensuring that even small farms can benefit from precision agriculture advancements (Tran et al., 2023).

Beyond subsidies, frugal innovation plays a key role in democratizing access to agricultural technologies. Low-cost phenotyping tools for African crops (Cudjoe et al., 2023) and smartphone-based farm management apps demonstrate how open-source and modular solutions can replace expensive proprietary systems. For example, East Africa’s M-Pesa mobile money platform enables microloans for farm inputs, reducing reliance on traditional banking systems that often exclude smallholders (Horgan et al., 2025; Naqvi et al., 2025). Similarly, Ethiopia’s digital soil mapping initiative, a collaboration between the government and telecom providers, delivers soil health data via SMS, helping farmers optimize fertilizer use at minimal cost (Naqvi et al., 2025). These innovations highlight how locally adapted; low-tech solutions can bridge the gap between advanced research and practical, on-ground implementation. Public-private partnerships (PPPs) further enhance accessibility by distributing costs and risks. Brazil’s FarmTech leasing programs, for instance, allow small farmers to use precision equipment without ownership costs, while India’s relaxed drone regulations (2021) have accelerated adoption by simplifying bureaucratic hurdles (Biradar and Hosalli, 2024). Additionally, regulatory reforms, such as Kenya’s lifting of its GMO ban (2022) and the EU’s evolving stance on gene-edited crops, demonstrate how policy flexibility can unlock new opportunities for smallholders (Horgan et al., 2025).

### 6.2 Technological and literacy gaps

The widespread implementation of precision agriculture technologies faces significant challenges due to digital illiteracy and technical skill gaps, particularly in rural and smallholder farming communities. Many advanced tools, such as drone-based crop monitoring, AI-driven pest detection (Yue et al., 2025), and automated livestock management systems (Bist et al., 2024), require a level of digital proficiency that remains out of reach for many farmers. A study in sub-Saharan Africa revealed that only 28% of smallholder farmers had received any training on digital farming tools, highlighting a critical barrier to adoption. Without proper education, farmers struggle to interpret data from soil sensors, satellite imagery, or machine learning-based advisories, leading to underutilization or misuse of these technologies (Naqvi et al., 2025).

Many precisions agriculture tools demand digital literacy and technical expertise that may not be readily available in rural communities. A study in sub-Saharan Africa found that only 28% of smallholder farmers had received any training on digital farming tools. Without proper education, farmers struggle to interpret data from soil sensors, drones, or AI-driven advisories, leading to suboptimal use of these technologies. Governments and NGOs are addressing this through farmer field schools (e.g., FAO’s Digital Villages Initiative) and simplified mobile apps with voice-based instructions in local languages. To bridge this gap, governments, NGOs, and aggrotech developers are implementing targeted capacity-building initiatives. The FAO’s Digital Villages Initiative, for example, trains farmers in low-income regions to use mobile-based agricultural advisories and sensor technologies through hands-on workshops. Similarly, simplified mobile apps with voice-based instructions in local languages are helping illiterate or semi-literate farmers access real-time farming insights without requiring advanced technical skills. In India, the Kisan Drones program not only subsidizes drone purchases but also includes mandatory training sessions to ensure farmers can operate them effectively (Biradar and Hosalli, 2024).

### 6.3 Policy and regulatory hurdles

- GMO Restrictions: Despite evidence of benefits, 32 countries (including EU member states) maintain strict bans or labeling requirements for genetically modified crops, stifling innovation. Despite scientific consensus on the safety and benefits of genetically modified crops, such as drought resistance and reduced pesticide use, 32 countries, including EU member states, enforce strict bans or mandatory labeling laws. These policies stifle agricultural innovation by limiting access to high-yielding or climate-resilient seed varieties. For example, while CRISPR-edited crops (which face fewer regulatory hurdles in some regions) show promise for smallholder farmers (Horgan et al., 2025), the EU’s precautionary approach continues to slow adoption. In contrast, Kenya’s 2022 decision to lift its GMO ban allowed the introduction of disease-resistant GM maize, demonstrating how policy shifts can unlock agricultural potential in food-insecure regions.
- Data Privacy Concerns: Farm data ownership disputes, such as who controls IoT-generated field data, have led to conflicts between farmers, aggrotech companies, and governments (Wiseman and Wright, 2022). In the EU, the General Data Protection Regulation (GDPR) imposes strict rules on agricultural data collection, sometimes discouraging farmers from adopting digital tools due to compliance burdens. Meanwhile, in the U.S., companies like John Deere have faced backlash for retaining ownership of machine-generated field data, limiting farmers’ autonomy. Recent frameworks, such as India’s 2023 Digital Agriculture Mission, attempt to balance innovation with farmer rights by mandating transparent data-sharing agreements (Orjuela-Garzon et al., 2024).
- Drones and Automation Laws: While drones offer transformative benefits, from pesticide spraying to crop health monitoring, bureaucratic delays in approvals slow their adoption. In India, despite the 2021 Kisan Drone subsidy scheme, complex licensing procedures persist. Similarly, Brazil’s restrictive flight permissions hinder large-scale drone deployments. However, countries like Zimbabwe and Rwanda have streamlined regulations, enabling rapid integration of drones for smallholder farming (Biradar and Hosalli, 2024).

### 6.4 Environmental and ethical risks

- E-Waste from IoT Devices: The rapid obsolescence of farm sensors and drones contributes to 53 million metric tons of global e-waste annually. Recycling programs remain underdeveloped.
- Monoculture Risks: Precision farming’s focus on maximizing yields can inadvertently promote single-crop systems, reduce biodiversity and increase pest vulnerability (Tscharntke et al., 2023).
- Energy Demands: Vertical farms and AI data centers require 3–5× more energy than traditional farming, raising sustainability questions unless powered by renewables (Avgoustaki and Xydis, 2022).

## 7 Future prospects & innovations

The future of agriculture is being reshaped by cutting-edge innovations that promise to enhance productivity, sustainability, and resilience in the face of climate change. Emerging technologies, from AI-driven predictive farming to blockchain-enabled supply chains, are unlocking new efficiencies, while the integration of renewable energy is making farming more sustainable. These advancements hold immense potential to address global food security challenges while mitigating agriculture’s environmental footprint.

### 7.1 Emerging trends in smart agriculture

- AI-Driven Predictive Farming: Artificial intelligence is evolving beyond real-time monitoring to anticipate agricultural risks before they occur. Machine learning models now analyze weather patterns, soil health trends, and pest migration data to forecast droughts, disease outbreaks, and optimal planting windows with 90%+ accuracy (IBM Research, 2023). Startups like Taranis use AI-powered drones to detect early signs of crop stress, enabling preemptive action.
- Blockchain for Transparent Supply Chains: Blockchain technology is improving food traceability and reducing fraud by recording every transaction, from farm to supermarket, on an immutable ledger. Walmart’s IBM Food Trust Network has reduced food recall times from 7 days to 2.2 seconds by tracking contaminated produce instantly (DeFranco et al., 2023). Similar systems are being adopted for fair-trade certification and carbon credit verification in agriculture.
- CRISPR & Next-Gen GMOs: Gene-editing tools like CRISPR-Cas9 are enabling the development of climate-resistant crops (e.g., flood-tolerant rice, heat-resistant wheat) without introducing foreign DNA, potentially bypassing regulatory hurdles faced by traditional GMOs (Waltz, 2016).

### 7.2 Integration with renewable energy

- Solar-Powered Smart Farms: Off-grid solar energy is powering IoT sensors, irrigation pumps, and vertical farms in remote regions. In sub-Saharan Africa, SunCulture’s solar drip irrigation has increased smallholder yields by 300% while cutting water and diesel costs (Burney et al., 2023).
- Agrivoltaics (Solar Farming + Crops): Dual-use solar farms, where crops are grown beneath elevated solar panels, are gaining traction. Trials show 40% higher land-use efficiency, with crops benefiting from partial shade (Adeh et al., 2022).
- Green Hydrogen for Fertilizer Production: Renewable hydrogen is being tested as a clean alternative to natural gas-based synthetic fertilizers, which account for 2% of global CO_2_ emissions.

### 7.3 Potential for global food security & climate resilience

- Precision Agriculture for Yield Gaps: AI and satellite-guided farming could close the 50% yield gap in developing countries by optimizing inputs (World Bank, 2023).
- Climate-Smart Crop Varieties: Drought-resistant maize and salt-tolerant soybeans are projected to reduce climate-related crop losses by 30% by 2030 (CGIAR, 2023).
- Decentralized Food Systems: Urban vertical farms and 3D-printed plant-based foods could reduce reliance on vulnerable supply chains.

### 7.4 Policy oriented implications and recommendation

Addressing the barriers to the adoption of modern agricultural technologies requires not only technological advancements but also supportive policy frameworks. Public-private partnerships (PPPs) are crucial in overcoming economic barriers to technology adoption. By combining the resources and innovation capabilities of the private sector with the regulatory and public interest focus of the government, PPPs can facilitate the development and dissemination of modern agricultural technologies. These partnerships can accelerate technology development by pooling funds for research and development initiatives that might otherwise be too costly or risky for individual entities. They also enhance the dissemination of technologies by leveraging private sector distribution channels and government-backed promotion initiatives. Furthermore, PPPs can improve access and training by developing and implementing programs that empower farmers with the necessary knowledge and skills to use new technologies effectively. Enabling policy frameworks are essential for creating an environment that supports the adoption of modern agricultural technologies. Subsidies and financial incentives can offset the high initial costs of new technologies, making them more accessible to farmers. Regulatory frameworks should be established to encourage innovation while ensuring safety and environmental standards are met. Access to finance can also be facilitated through policies that make it easier for farmers to obtain credit and financial services, helping them invest in new technologies without being burdened by prohibitive upfront costs.

## 8 Conclusion

The integration of modern agricultural technologies presents a transformative opportunity to address the dual challenges of global food security and environmental sustainability, yet their widespread adoption requires coordinated efforts across multiple sectors. Key findings from this comprehensive review demonstrate that precision farming techniques can increase crop yields by 20-50% while reducing input waste, with vertical farming systems achieving up to 95% improvements in land and water use efficiency. Biotechnology innovations, particularly drought-resistant and pest-resistant crop varieties, have shown remarkable success in stabilizing yields under climate stress, while AI-driven predictive analytics are revolutionizing farm management through real-time decision support. However, significant barriers persist, including prohibitive upfront costs for smallholder farmers, with autonomous equipment ranging from $300,000-$500,000 and IoT sensor networks requiring $500-$2,000 per hectare, creating a pressing need for innovative financing models and public-private partnerships. Policy frameworks must evolve to address regulatory bottlenecks surrounding GMO adoption, data privacy concerns, and drone usage restrictions, while simultaneously investing in digital literacy programs to bridge the technological skills gap prevalent in rural communities. Environmental considerations remain paramount, as the agricultural sector must balance technological adoption with sustainable practices to prevent unintended consequences such as soil microbiome disruption from over-automation or e-waste accumulation from obsolete devices. Looking ahead, the most promising pathway involves a synergistic approach combining precision technologies with regenerative agricultural principles, supported by targeted policy interventions, increased R&D funding, and inclusive implementation strategies that prioritize smallholder farmers. This balanced adoption framework, coupled with renewable energy integration and circular economy principles for aggrotech equipment, can position modern agriculture as a cornerstone of both global food systems and climate change mitigation efforts in the coming decades.
